# Risk factors for sudden cardiac death in heart failure: A systematic review and meta-analysis of risk factors derived from prediction models

**DOI:** 10.1097/MD.0000000000050378

**Published:** 2026-08-28

**Authors:** Chenggong Xie, Jinrui Huang, Zhijia Xiang, Zhao Wang, Yun Long, Bin Wang, Hao Liang

**Affiliations:** a Institute of Information on Traditional Chinese Medicine, China Academy of Chinese Medical Sciences, Beijing, China; b School of Informatics, Hunan University of Chinese Medicine, Changsha, Hunan, China; c School of Nursing, Hunan University of Chinese Medicine, Changsha, Hunan, China; d Academy of Chinese Medical Sciences, Hunan University of Chinese Medicine, Changsha, Hunan, China; e Cardiovascular Department, The First Hospital of Hunan University of Chinese Medicine, Changsha, Hunan, China.

**Keywords:** heart failure, meta-analysis, risk factors, risk prediction models, sudden cardiac death

## Abstract

**Background:**

Heart failure (HF) is a global health burden with high mortality. Sudden cardiac death (SCD) remains a major complication, highlighting the need for accurate risk prediction.

**Methods:**

We conducted a systematic review and meta-analysis, guided by the Population, Intervention, Comparator, Outcome, Timing, and Setting framework, to identify risk factors for SCD in HF and assess prediction models. Searches across 8 databases yielded eligible studies. Data extraction, risk of bias assessment using the Prediction Model Risk of Bias Assessment Tool, and statistical analyses were performed.

**Results:**

Twelve studies met inclusion criteria, with 8 included in the meta-analysis. New York Heart Association classification and left ventricular ejection fraction emerged as the most robust predictors of SCD. Additional significant factors included age, sex, ischemic etiology, diabetes, heart rate, sodium, potassium, creatinine, estimated glomerular filtration rate, and hemoglobin. Considerable heterogeneity was observed among studies.

**Conclusion:**

New York Heart Association class and left ventricular ejection fraction are key predictors of SCD in HF, while demographic, etiological, and laboratory factors further refine risk assessment. Current models show limitations due to heterogeneity and lack of external validation. Future work should integrate refined predictors, treatment responses, and diverse populations to improve the accuracy and clinical utility of SCD risk stratification in HF.

## 1. Introduction

Heart failure (HF) remains a major global health challenge, with persistently high mortality and hospitalization rates despite advancements in treatment and management. While novel therapies and devices have shown potential in mitigating some aspects of the disease, the overall impact on mortality remains limited, and the burden on healthcare systems continues to grow.^[1]^ Among the complications of HF, sudden cardiac death (SCD) is a leading cause of mortality, often occurring unexpectedly in both asymptomatic patients and those with recent symptom onset. SCD accounts for approximately 25% of the 17 million cardiovascular deaths globally each year.^[2,3]^ This underscores the urgent need to better understand the risk factors associated with SCD in HF patients and to improve strategies for its prediction and prevention.^[4]^

SCD in HF is a multifactorial event, with numerous clinical, laboratory, and demographic factors contributing to risk. While current research has identified a range of potential predictors, including biomarkers, clinical characteristics, and advanced imaging findings such as magnetic resonance imaging,^[5,6]^ the mechanisms behind SCD remain complex and not fully understood. Moreover, the development of accurate and reliable risk prediction models is hindered by challenges such as small sample sizes, inconsistent methodologies, and a lack of external validation.^[7]^ The interplay between various risk factors further complicates the accurate prediction of SCD, leading to variability in research findings.

The need for better-defined risk prediction models is critical, particularly for guiding clinical decision-making regarding interventions such as implantable cardioverter-defibrillators (ICDs), which can significantly improve outcomes in high-risk HF patients. This systematic review and meta-analysis aims to address these gaps by synthesizing the available evidence on risk factors for SCD in HF and assessing the performance of existing prediction models. Through a comprehensive evaluation of the available data, we aim to clarify the key predictors of SCD in this population and provide insights into the refinement of clinical risk assessment strategies.

## 2. Materials and methods

This study protocol was registered on the International Prospective Register of Systematic Reviews (registration number: CRD42024506527). All analyses were based on previously published studies; thus, no ethical approval and patient consent were required.

### 2.1. Search strategy

A comprehensive search strategy was designed and executed within PubMed, Web of Science, Embase, The Cochrane Library, Google Scholar, Sinomed, China National Knowledge Infrastructure, and Wanfang databases from their establishment until February 15, 2024. The search strategy was developed through a combination of subject terms and keywords, with adjustments based on the differences among various databases. The following words were used: “Heart Failure,” “Sudden Cardiac Death,” “Risk Assessment,” “Risk Prediction,” “Risk Model,” and “Risk Score.” The detailed search strategies used for the study can be found in Table S1, Supplemental Digital Content 1.

In this study, we rigorously adhere to the Population, Intervention, Comparator, Outcome, Timing, and Setting (PICOTS) framework to refine our inclusion and exclusion criteria for meta-analysis. By systematically applying the PICOTS framework, we ensure a structured and transparent approach to study selection, enhancing the rigor and comprehensiveness of our analysis methods. Outlined below are the specific principles of the PICOTS framework:

P (Population): Patients with HF at risk of SCD.
I (Intervention model): Risk prediction models for SCD in patients with HF that have been developed and published (predictors ≥ 3).
C (Comparator): No competing model.
O (Outcome): The outcome focuses on the risks of SCD in patients with HF.
T (Timing): The outcome is predicted after evaluating basic patient demographics, medical history, cardiac function parameters, and relevant diagnostic tests.
S (Setting): The intended use of the risk prediction model is to individualize the prediction of SCD in patients with HF, aiding in the implementation of appropriate preventive strategies and interventions to mitigate adverse events.

### 2.2. Inclusion and exclusion criteria

The inclusion criteria for our meta-analysis were as follows: studies involving patients diagnosed with HF at risk of SCD; studies reporting on the development or validation of risk prediction models for SCD; and studies with SCD as the primary outcome of interest.

Conversely, the exclusion criteria encompassed: studies that did not develop or validate a risk prediction model for SCD; studies with outcomes limited to subgroups of SCD; studies not available in English or Chinese; studies for which full-text access could not be obtained despite attempts to contact authors via email; and studies where the risk prediction model did not report precise hazard ratios (HRs).

### 2.3. Study selection

The study selection process was carried out independently by 3 researchers (CGX, ZJX, and ZW). Initially, duplicate studies were identified and removed through systematic screening. Subsequently, the remaining studies underwent initial screening based on their titles and abstracts to assess their eligibility according to the predefined inclusion and exclusion criteria. Following this initial screening, the full texts of potentially eligible studies were reviewed by each researcher. In addition, an examination of the reference lists of all eligible studies was conducted to ensure the comprehensive identification of all relevant studies during the initial search. Consensus discussions involving all 3 researchers were used to resolve any discrepancies or disagreements regarding study selection.

### 2.4. Data extraction

Data extraction was independently conducted by 3 researchers (CGX, ZJX, and ZW), following a standardized protocol. Any discrepancies in data extraction were resolved through discussion among the researchers or by consulting a fourth reviewer. The extracted information from the selected studies was organized into 2 main categories: basic information, which included details such as the author, year of publication, study design, participants, data source, main death outcome, included predictive factors, and sample size; model information, which encompassed various aspects related to the prediction model, including model development method, model variable method, model performance, and model presentation. To ensure accuracy and consistency across all included studies, the initial extraction of information was conducted by one researcher, with a subsequent review and cross-check of the extracted data by another researcher.

### 2.5. Quality assessment

To assess the quality and potential bias risk of the included studies, we employed the Prediction Model Risk of Bias Assessment Tool (PROBAST). This tool facilitated the evaluation of bias and concerns regarding the applicability of the included studies through the systematic application of the PROBAST checklist.

Two researchers (ZW and ZJX) assessed the presence of bias and applicability concerns within the studies using the PROBAST checklist. The PROBAST checklist is a comprehensive tool for quality assessment, designed to evaluate studies involved in the development, validation, or updating of prediction models. It consists of 20 questions categorized into 4 domains: Participants, Predictors, Outcome, and Analysis. Each signaling question can be answered with 1 of 5 responses: “yes,” “probably yes,” “no,” “probably no,” or “no information.” If 1 or more questions within a domain are answered as “no” or “probably no,” the domain is considered to have a high risk of bias. Conversely, should all domains be deemed to have a low risk of bias, the overall bias is considered low; otherwise, it is categorized as high risk. This systematic approach ensures thorough quality assessment and transparency in evaluating the risk of bias across studies.

### 2.6. Data synthesis and statistical analysis

A meta-analysis was conducted to combine HR extracted from the included studies using R version 4.2.1 (R Foundation for Statistical Computing, Vienna, Austria, meta packages). Heterogeneity among the studies was assessed using the *I*^2^ statistic and Cochran’s *Q* test. In this study, the *I*^2^ statistic quantifies the proportion of total variation across studies attributed to heterogeneity, providing a numerical measure of this variability. A value of *I*^2^ <50% is indicative of low heterogeneity, thus warranting the adoption of a fixed-effects model for analysis. Conversely, an *I*^2^ value exceeding 50% signifies high heterogeneity, necessitating the application of a random-effects model to accommodate this variability. This methodological approach aligns with established standards in meta-analysis, ensuring consistent and accurate estimation of pooled effect sizes across studies.

Publication bias was not formally assessed because fewer than 10 studies were available for each pooled analysis, making funnel plots and Egger regression test unreliable.

## 3. Results

### 3.1. Study selection

**Figure 1.**
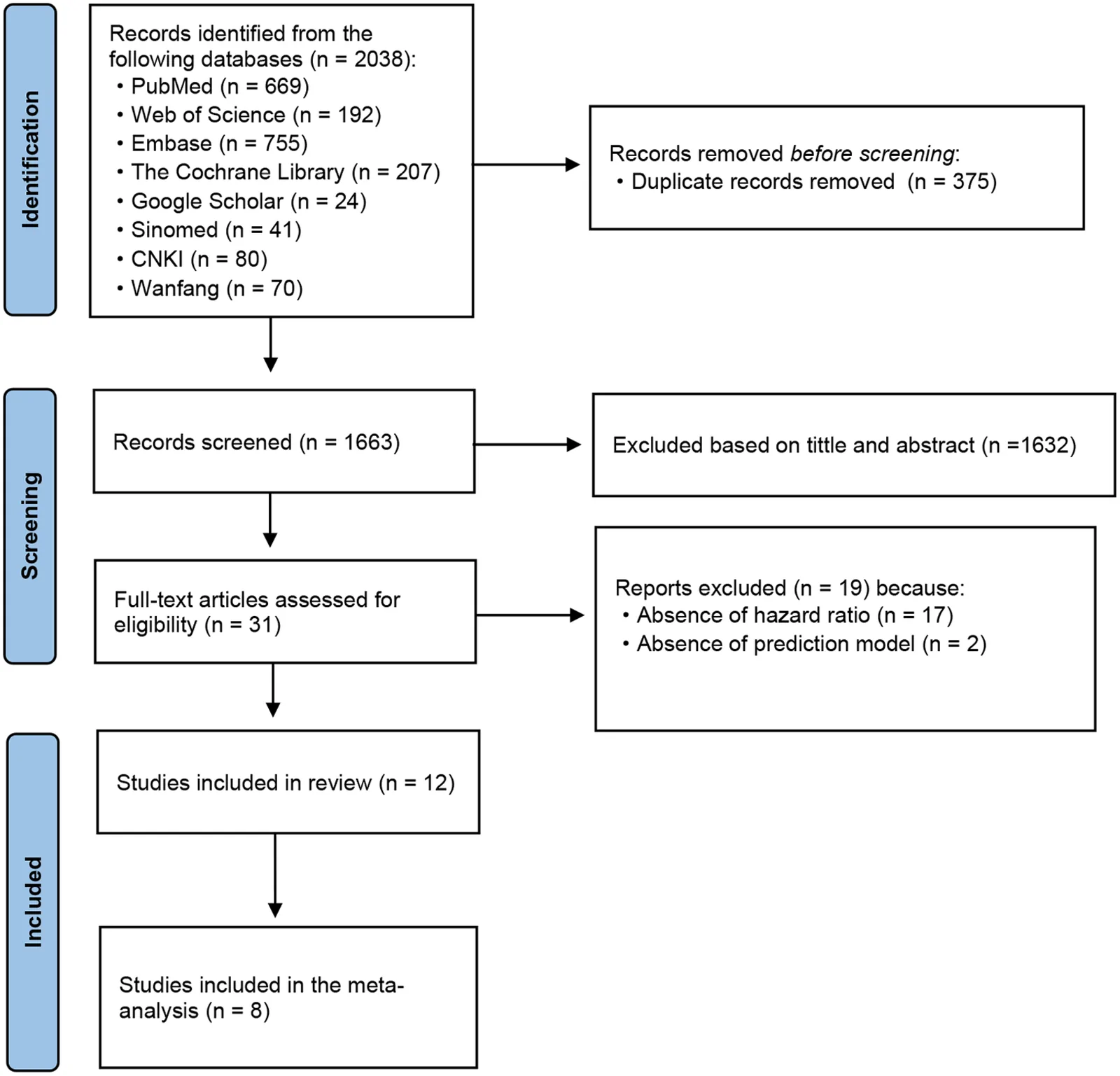
Preferred Reporting Items for Systematic Reviews and Meta-Analyses flowchart of literature search and selection.

The Preferred Reporting Items for Systematic Reviews and Meta-Analyses 2020 flowchart, depicted in Figure 1, illustrates the rigorous and systematic approach undertaken in our study for literature selection. This comprehensive process involved searching 8 databases, screening titles and abstracts, assessing full-text of potentially relevant studies, and subsequently including eligible studies based on predefined criteria.

The literature search process commenced with an initial retrieval of 2038 indexed records. Following the removal of 375 duplicate records, we screened 1663 titles and abstracts to determine eligibility. Subsequently, 31 articles were selected for further evaluation. Among these, 19 studies were excluded from the review; reasons for exclusion included 17 studies lacking HR values and 2 studies lacking a risk prediction model. Ultimately, 12 articles were deemed eligible for inclusion in the review,^[8–19]^ out of which 8 underwent meta-analysis.^[8,10–13,15,17,18]^

### 3.2. Study characteristics and model validation

Table 1 presents baseline data from the 12 studies included in the review, published between 2006 and 2019. Regarding the study population, variations across studies were observed in terms of age, gender, ischemic etiology, diabetes mellitus, heart rate, sodium, potassium, creatinine, estimated glomerular filtration rate (eGFR), hemoglobin, New York Heart Association (NYHA) classification, and left ventricular ejection fraction (LVEF).

**Table 1:** Overview of basic data of the included studies.

Author (yr)	Country	Study design	Participants	Diagnostic criteria	Data source	Main death outcome	Included predictive factors in meta-analysis	SCD cases/sample size (%)
Julia Ramirez (2017)	United Kingdom	Prospective cohort study	Patients ≥18 yr old with symptomatic CHF corresponding to NYHA classes II and III	HF: A SCD: E	A multicenter study of 8 university hospitals	SCD, PFD	NYHA class III, LVEF ≤ 35%	49/597 (8.2%)
Iyo Ikeda-Yorifuji (2019)	Japan	Prospective cohort study	Patients with HFrEF corresponding to LVEF ≤ 40%	HF: C SCD: E	Outpatients of a hospital	SCD	LVEF ≤ 40%	22/90 (24.4%)
Luis Rohde (2021)	United States of America	Prospective cohort study	Patients with CHF corresponding to NYHA classes II–IV and LVEF ≤ 40%	HF: C SCD: E	Outpatients of a hospital	SCD, CVD, non-CVD	NYHA class III and IV	93/8399 (1.1%)
Andrew Xanthopoulos (2023)	Greece	Prospective cohort study	Patients ≥20 yr old with AHF	HF: D SCD: F	A multicenter, observational study of REALITY-AHF	SCD, heart failure deaths, other cardiovascular deaths	–	23/284 (8.1%)
Poole-Wilson (2015)	United Kingdom	Prospective cohort study	Patients with HF corresponding to NYHA classes II–IV	HF: C SCD: F	An international multicentre trial of lisinopril in patients with chronic heart failure	CVD, non-CVD, death of unknown cause	–	589/3164 (18.6%)
Alexander Vladimirovich Frolov (2017)	Belarus	Prospective cohort study	Patients ≥38 yr old with symptomatic CHF corresponding to LVEF ≤ 44%	HF: D SCD: F	Inpatients of a hospital	SCD, VTA	LVEF ≤ 21%	9/240 (3.8%)
Josep Lupón (2019)	Spain	Prospective cohort study	Patients ≥55 yr old with HF corresponding to LVEF ≤ 50%	HF: D SCD: E	Outpatients of a hospital	HF, SCD	NYHA class III and IV, LVEF < 45%	40/744 (5.4%)
Selcuk Adabag (2014)	United Kingdom	Prospective cohort study	Patients ≥60 yr old with symptomatic HF corresponding to NYHA classes II–IV and EF ≥ 45%	HF: B SCD: E	Inpatients and outpatients of a hospital	SCD, other types of death	NYHA class II and III	231/4128 (5.6%)
Tariq Ahmad (2014)	United States of America	Prospective cohort study	Patients with CHF corresponding to LVEF < 35%	HF: C SCD: E	Inpatients of a hospital	SCD, pump failure, other types of death	–	42/813 (5.2%)
Meena Rao (2017)	United Kingdom	Prospective cohort study	Patients with STICH corresponding to LVEF ≤ 35%	HF: C SCD: E	Inpatients of a hospital	SCD, other types of death	–	113/1411 (8.0%)
Ronald Freudenberger (2007)	United Kingdom	Prospective cohort study	Patients with HF corresponding to NYHA classes II and III and LVEF ≤ 35%	HF: C SCD: F	Inpatients of a hospital	Stroke, peripheral embolism, pulmonary embolism, any thromboembolic event	LVEF ≤ 35%	85/2521 (4.0%)
Leslie Saxon (2006)	United Kingdom	Prospective cohort study	Patients with HF corresponding to NYHA classes III–IV and LVEF ≤ 35%	HF: C SCD: E	Inpatients of a hospital	SCD	NYHA class IV, LVEF > 20%	83/1520 (5.5%)

“–” = not reported, A = types of heart failure with preserved and decreased ejection fraction, AHF = acute heart failure, B = types of heart failure with preserved ejection fraction, C = types of heart failure with decreased ejection fraction, CHF = congestive heart failure, CVD = cardiovascular disease, D = unclear definition of heart failure types, E = well-defined sudden cardiac death, EF = ejection fraction, F = unclear definition of sudden cardiac death, HF = heart failure, HFrEF = heart failure with reduced ejection fraction, LVEF = left ventricular ejection fraction, NYHA = New York Heart Association, PFD = pump failure death, REALITY-AHF = registry focused on very early presentation and treatment in emergency department of acute heart failure, SCD = sudden cardiac death, STICH = the Surgical Treatment of Ischemic Heart Failure, VTA = ventricular tachyarrhythmias.

Table 2 provides a detailed overview of the models utilized in the 12 studies included in this analysis. Specifically, it includes information on the author (year), model development method, model variable method, model performance (HR and 95% confidence interval [CI]), and model presentation.

**Table 2:** Overview of the information of the included prediction models.

Author (yr)	Model development method	Model variable method	Model performance (HR and 95% CI)		Model presentation	
			NYHA	LVEF		
Julia Ramirez (2017)	Integrated risk model	A, B	2.19 (1.18–4.07)	2.34 (1.24–4.40)		ROC curves
Iyo Ikeda-Yorifuji (2019)	Cox regression analysis	A, B	1.41 (0.66–3.02)	0.95 (0.90–1.00)		SCD-free rate and severe arrhythmic event-free rate curves
Luis Rohde (2021)	Time-updated multivariable-adjusted cox models, CART, logistic regression analysis	A	1.53 (1.19–1.95)	–		Plot of cause-specific hazard ratios from cox survival analysis
Andrew Xanthopoulos (2023)	Fine–Gray competing risk regression	B	–	–		Cumulative incidence curves
Poole-Wilson (2015)	Competing risks analysis	A	–	–		Competing risks analysis HR
Alexander Vladimirovich Frolov (2017)	Cox logistic regression analysis	A, B	–	2.43 (1.21–5.02)		Probability values of adverse cardiovascular events
Josep Lupón (2019)	Fine–Gray method of cox regressions analysis	A, B	1.72 (0.91–3.24)	2.48 (0.97–6.33)		Cumulative incidence curves
Selcuk Adabag (2014)	Cox regression analysis	A, B	1.06 (0.77–1.46)	–		Cumulative incidence curves
Tariq Ahmad (2014)	Cox regression analysis	B	–	–		Cumulative incidence curves, Kaplan–Meier survival curves
Meena Rao (2017)	Cox regression analysis	A, B	–	–		Cumulative incidence curves
Ronald Freudenberger (2007)	Cox regression analysis	B	–	0.82 (0.69–0.97)		Proportion of patients with thromboembolic event
Leslie Saxon (2006)	Cox regression analysis	B	2.62 (1.61–4.26)	0.55 (0.35–0.87)		Kaplan–Meier survival curves

“–” = not reported, A = univariate analysis, B = multivariate analysis, CART = classification and regression tree, HR = hazard ratio, LVEF = left ventricular ejection fraction, NYHA = New York Heart Association, ROC = receiver operating characteristic, SCD = sudden cardiac death.

### 3.3. Quality assessment

Table S2, Supplemental Digital Content 2 summarizes the study types, results of bias risk, and applicability, assessed using PROBAST for the 12 included studies. The PROBAST checklist utilized in this study is recognized as an authoritative instrument for assessing prediction models. The results reveal that among the 12 studies, 11 solely developed models without external validation, while only 1 study completed external validation. Regarding the risk of bias and applicability, only 1 study demonstrated a low risk of bias, whereas 11 studies exhibited a medium or high risk of bias. In addition, 7 studies displayed low applicability, whereas 5 studies showed medium or high applicability. Most studies lacked sufficient disclosure of model details and external validation, potentially introducing uncertainty or inherent risks to the models. Therefore, future research endeavors should emphasize the disclosure of more detailed information and incorporate external validation to ensure the applicability and reliability of model effect estimates.

### 3.4. Meta-analysis for the main risk factors of the included models

**Figure 2.**
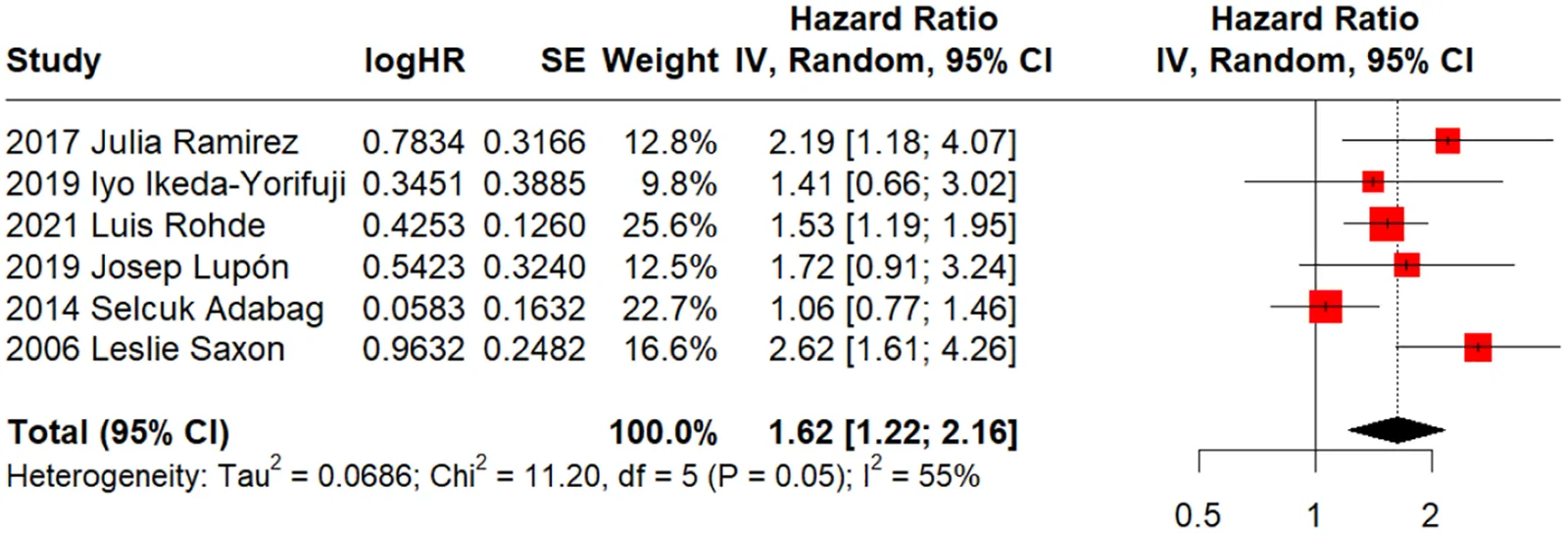
Forest plot of the random-model meta-analysis of pooled hazard ratio for New York Heart Association. CI = confidence interval, HR = hazard ratio.

Due to insufficient disclosure of relevant details in some studies, only 8 studies were ultimately included in the meta-analysis. Finally, NYHA classification and LVEF were identified as the main risk factors of the included models. For NYHA classification, a total of 6 studies were included in the synthesis, yielding a pooled HR of 1.62 (95% CI = 1.22–2.16; Fig. 2).

**Figure 3.**
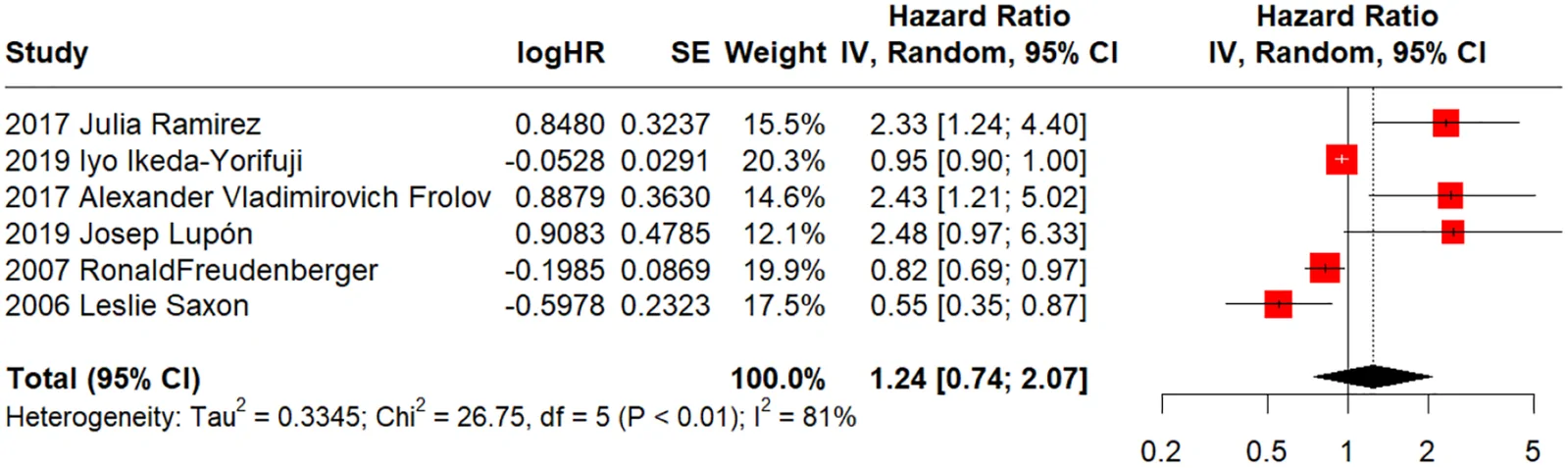
Forest plot of the random-model meta-analysis of pooled hazard ratio for left ventricular ejection fraction. CI = confidence interval, HR = hazard ratio.

Similarly, for LVEF, 6 studies were synthesized, resulting in a pooled HR of 1.24 (95% CI = 0.74–2.07; Fig. 3).

The *I*^2^ values for the 2 meta-analyses were 55% and 81%, respectively, indicating high heterogeneity among the included studies. To explore potential sources of heterogeneity, subgroup analyses were conducted based on follow-up durations (48 and 44 months); however, the *I*^2^ values remained high in both cases, suggesting that follow-up duration may not be a major contributor to the observed heterogeneity (Figs. S1 and S2, Supplemental Digital Content 3).

### 3.5. Meta-analysis for the other risk factors of the included models

Due to a limited number of studies addressing certain risk factors, we only included those with 3 or more studies for meta-analysis. Apart from the main risk factors for NYHA classification and LVEF, information pertaining to other risk factors’ meta-analyses (including HR values, *I*^2^ values, statistical methods, and *P* values) has been compiled into Table S3, Supplemental Digital Content 4.

## 4. Discussion

SCD stands as one of the prominent causes of mortality globally.^[20]^ To address this challenge, ICDs are frequently employed in clinical practice to play a pivotal role in preventing SCD.^[21]^ Given the established efficacy of ICDs as a primary preventive measure for SCD, the accurate prediction of SCD risk holds substantial clinical significance and urgency.^[22]^

Currently, LVEF serves as the primary biomarker for predicting SCD, yet relying solely on it in clinical practice yields suboptimal outcomes.^[23]^ This underscores the inadequacy of using LVEF alone as a predictive marker for primary prevention, particularly considering that most patients who receive ICDs may not benefit from it.^[24]^ Therefore, there is a pressing need to obtain more risk prediction factors beyond LVEF.

Of particular concern is the implementation of Personalised Risk Prediction and Prevention of Sudden Cardiac Death After Myocardial Infarction study, which demonstrated that using a fixed ejection fraction threshold of 35% to determine whether a patient should receive an ICD is inherently flawed.^[25]^ The challenge lies in identifying how to avoid unnecessary ICD implantation in patients with an ejection fraction of ≤35%, while ensuring appropriate protection for patients with an ejection fraction >35%. The Personalised Risk Prediction and Prevention of Sudden Cardiac Death After Myocardial Infarction study clearly indicates the need for a novel approach to address this issue.

Accurately assessing individual patients’ risk of SCD and implementing preventive measures targeting modifiable risk factors are critical strategies for mitigating the burden of SCD.^[26]^ The present systematic review and meta-analysis aimed to elucidate the risk factors associated with SCD in HF patients, as well as to evaluate the performance of existing risk prediction models.

A total of 6 countries were represented in the baseline data of the included studies, with 6 studies from the United Kingdom, 2 from the United States of America, and 1 each from Japan, Greece, Belarus, and Spain. Variability in populations and lifestyles across different countries may contribute to inconsistencies in results. Importantly, all studies were prospective cohort studies. Regarding data sources, 10 studies were sourced from hospitals and outpatient departments, with only 2 studies originating from clinical trials, ensuring the reliability of basic patient data. Ten studies had 2 or more primary mortality outcomes, with only 2 studies having SCD as the primary outcome. The diversity in mortality outcomes may influence interactions between different risk factors.^[27]^ Regarding the threshold settings for risk factors, each study had different thresholds, which could affect the final effect estimates and may also contribute to the high heterogeneity observed in the meta-analysis. In addition to differences in predictor thresholds, several methodological factors may have contributed to the substantial heterogeneity observed across studies. First, the included studies enrolled heterogeneous HF populations, including patients with heart failure with reduced ejection fraction, heart failure with preserved ejection fraction, acute HF, and mixed HF cohorts, which may differ substantially in the underlying mechanisms and incidence of SCD. Second, the definition of SCD was not completely consistent across studies, with some studies using well-defined adjudicated SCD outcomes whereas others adopted broader or less explicit definitions. Third, different statistical modeling strategies were applied, including conventional Cox proportional hazards models, competing-risk models, and integrated prediction models, which may yield different effect estimates. Finally, only 1 study performed external validation, whereas most prediction models were developed without independent validation, potentially limiting their generalizability and increasing between-study variability. The majority of studies collected data from hospitals, with only 3 relying on data from existing studies. And the proportion of SCD samples to total included samples ranged from 1.1% to 24.4% across studies, with 9 studies having this proportion concentrated between 3% and 9%.

In the information regarding the included prediction models from the 12 studies, concerning the model development method, 7 studies employed the Cox regression analysis model, 2 studies utilized competing risks analysis, while the remaining studies employed various methods such as integrated risk models and Cox logistic regression analysis. Only 1 study utilized a multi-model approach, constructing time-updated multivariable-adjusted Cox models, classification and regression tree, and logistic regression analysis. Regarding the model variable method, 2 studies solely utilized univariate analysis, 4 studies exclusively utilized multivariate analysis, while the remaining 6 studies employed a combination of univariate and multivariate analysis. Differences in model development and model variables may result in subtle variations in the final results, with existing research indicating that standard Cox models may overestimate the risk of SCD compared to competing risk models.^[28]^ In terms of model presentation, 5 studies employed cumulative incidence curves, 2 studies utilized Kaplan–Meier survival curves, while the presentation of results varied across the remaining studies.

Our analysis underscores the importance of risk factors such as NYHA classification and LVEF in predicting the risk of SCD among HF patients. Furthermore, we observed that age, gender, ischemic etiology, diabetes mellitus, heart rate, sodium, potassium, creatinine, eGFR, and hemoglobin – comprising a total of 10 risk factors – are associated with the risk of SCD in HF patients.

The NYHA classification, widely employed to assess the functional status of HF patients, emerged as a significant predictor of SCD risk in our meta-analysis. In the meta-analysis, NYHA demonstrated a HR of 1.62 (95% CI = 1.22–2.16), indicating a direct association between NYHA classification and SCD risk in HF patients, with higher NYHA levels correlating with increased SCD risk. Specifically, patients classified as NYHA class III or IV face notably elevated SCD risk compared to those in NYHA class I or II, reflecting the progressive nature of HF and the cumulative burden of cardiac dysfunction and hemodynamic instability. This finding contrasts with previous studies, which suggested no difference in SCD risk between NYHA class I and NYHA class II-III patients.^[29]^

LVEF, a critical parameter for assessing cardiac function, plays a central role in risk stratification and prognosis determination. HF patients with reduced LVEF, typically defined as LVEF ≤ 40%, face significantly elevated risks of adverse outcomes, including SCD. Although LVEF remains one of the most widely used clinical indicators for SCD risk stratification and ICD decision-making, the pooled estimate in our meta-analysis did not reach statistical significance (HR = 1.24, 95% CI = 0.74–2.07). Therefore, our findings do not provide sufficient evidence to confirm an independent association between LVEF and SCD risk. Studies have shown that the use of standard LVEF-based criteria has led to many HF patients at risk of SCD receiving ICDs.^[30]^ However, identifying candidates for ICD therapy among HF patients with low LVEF remains a key clinical challenge for determining the appropriate application of ICDs.^[31]^

Beyond estimating the risk of SCD, risk prediction models should play a central role in guiding individualized therapeutic decision-making. In contemporary HF management, one of the primary objectives of risk stratification is to identify patients most likely to benefit from ICD implantation while avoiding unnecessary device therapy in patients with low arrhythmic risk. Therefore, future prediction models should move beyond single-risk estimation and support precision treatment strategies tailored to individual patient profiles.

Emerging evidence suggests that inflammatory and metabolic biomarkers provide additional prognostic information beyond traditional clinical characteristics. For example, serum albumin has been associated with adverse outcomes in patients receiving cardiac resynchronization therapy, reflecting the influence of nutritional status and systemic inflammation on prognosis.^[32]^ Similarly, the triglyceride-glucose index, a surrogate marker of insulin resistance and metabolic dysfunction, has been shown to predict long-term mortality and appropriate ICD therapy in patients with HF.^[33]^ These findings indicate that incorporating inflammatory and metabolic biomarkers into future prediction models may further improve individualized risk assessment.

In addition, recent studies have proposed several prognostic markers and mortality prediction scores specifically for patients undergoing ICD implantation. These studies suggest that combining conventional clinical characteristics with systemic inflammatory indices and validated mortality prediction scores may improve the identification of patients who derive the greatest survival benefit from device therapy.^[34–36]^ Such multidimensional approaches may also reduce unnecessary ICD implantation in patients with limited expected benefit.

In terms of demographic factors, the HR for age and gender were 1.05 and 1.87, respectively, confirming that the risk of SCD increases with age and is higher in male patients.^[37,38]^ Regarding etiology, the HR for diabetes mellitus and ischemic causes were 1.61 and 1.57, respectively. While HF patients themselves have a relatively low risk of SCD, the presence of diabetes mellitus significantly elevates this risk.^[39]^ Similarly, the coexistence of ischemic conditions, such as myocardial infarction, increases the likelihood of ventricular arrhythmias and raises the risk of SCD.^[40]^

In clinical and laboratory indicators, the HR for creatinine, heart rate, and potassium were 1.97, 1.02, and 1.53, respectively, while those for eGFR, hemoglobin, and sodium were 0.98, 0.89, and 0.96, respectively. Creatinine and eGFR reflect renal function status and are inversely correlated. Chronic kidney disease elevates the risk of both HF and SCD, with early increases in creatinine and normal or increased eGFR values, while persistent increases in creatinine and decreased eGFR values occur in later stages.^[41]^

Heart rate and heart rate variability are moderately associated, but the role of heart rate variability in predicting SCD risk in HF patients remains controversial. While some studies suggest that heart rate variability cannot predict SCD risk, further research is warranted in this area.^[42,43]^ Sodium and potassium ions are closely related to action potentials, and prolonged action potential duration is a significant contributor to SCD risk in HF patients.^[44]^ Hemoglobin levels determine the occurrence and severity of anemia in HF patients, with lower hemoglobin levels associated with increased anemia severity and higher SCD risk.^[45]^

## 5. Limitations

This meta-analysis has several limitations. First, only studies published in English or Chinese were included; therefore, relevant studies published in other languages may have been missed, introducing potential language bias. Second, studies that did not report HRs or sufficient data for HR estimation were excluded from the quantitative synthesis. Although this approach ensured methodological consistency, it may have introduced selection bias by excluding potentially informative prediction models. Third, substantial heterogeneity remained across several pooled analyses. This heterogeneity may reflect differences in HF phenotypes, definitions of SCD, predictor thresholds, statistical modeling strategies, and the limited use of external validation among the included studies. Because only 8 studies were eligible for quantitative synthesis, further subgroup analyses based on these characteristics were not statistically robust. Finally, treatment strategies differed across studies, including pharmacological therapy, ICD implantation, and other device-based interventions. The inconsistent reporting of treatment information prevented us from evaluating their influence on SCD risk or incorporating treatment response into the pooled analyses.

## 6. Conclusions

This study underscores the clinical significance of accurately predicting SCD risk in HF patients. The systematic review included a total of 12 studies, with 8 studies included in the meta-analysis. Through a comprehensive assessment of the included studies, 12 risk factors were identified for predicting SCD risk in HF patients. Among these, NYHA classification and LVEF emerged as crucial predictive risk factors, with combined HR values of 1.62 (95% CI = 1.22–2.16) and 1.24 (95% CI = 0.74–2.07), respectively. In addition, demographic factors, etiology, and clinical/laboratory indicators, including age, gender, ischemic etiology, diabetes mellitus, heart rate, sodium, potassium, creatinine, eGFR, and hemoglobin, also played crucial roles, as detailed in Table S3, Supplemental Digital Content 4.

However, according to the PROBAST evaluation of the 12 models, 11 studies lacked external validation and exhibited medium to high bias risk, while 5 studies demonstrated medium to high applicability. The majority of studies did not meet PROBAST requirements, highlighting the necessity for future researchers to incorporate external validation into their models to improve transportability and generalizability. Moreover, disclosing more details about the models in studies would enhance the quality of future research.

Future efforts should prioritize the development of multicenter, externally validated, highly transparent models with rigorous experimental designs and diverse samples representing various populations and ethnicities. This approach will better suit the needs of clinical patients in the real world.

## Author contributions

**Data curation:** Chenggong Xie, Jinrui Huang, Zhijia Xiang, Zhao Wang.

**Methodology:** Chenggong Xie.

**Formal analysis:** Jinrui Huang.

**Funding acquisition:** Bin Wang, Hao Liang.

**Writing – original draft:** Chenggong Xie, Yun Long, Bin Wang, Hao Liang.

**Writing – review & editing:** Chenggong Xie, Yun Long, Bin Wang, Hao Liang.










