# Supplementary material for: Risk factors for sudden cardiac death in heart failure: A systematic review and meta-analysis of risk factors derived from prediction models
Source: Medicine (Baltimore). 2026 Aug 28;105(35):e50378. doi: 10.1097/MD.0000000000050378 (PMC13529164; doi:10.1097/MD.0000000000050378)
Supplement: Supplementary file 1 [file medi-105-e50378-s003.pdf]

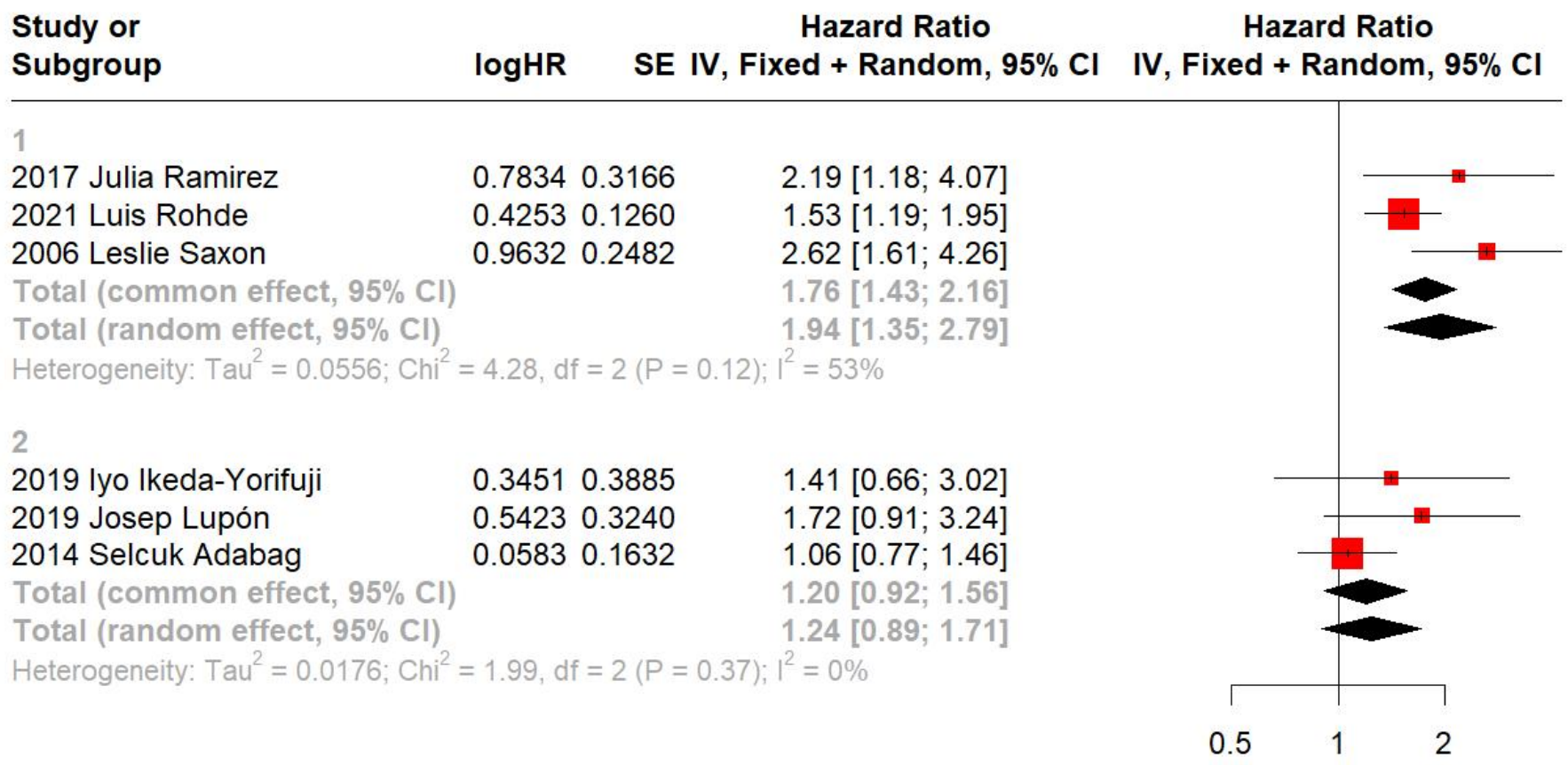

Supplemental Figure 1. Forest plot of the fixed and random model subgroup analysis for New York Heart Association.
