# Supplementary material for: Risk factors for sudden cardiac death in heart failure: A systematic review and meta-analysis of risk factors derived from prediction models
Source: Medicine (Baltimore). 2026 Aug 28;105(35):e50378. doi: 10.1097/MD.0000000000050378 (PMC13529164; doi:10.1097/MD.0000000000050378)
Supplement: Supplementary file 3 [file medi-105-e50378-s001.doc]

Supplemental Table 1. The detailed search strategies of the study.

| Database | Search Strategy | Results |
| --- | --- | --- |
| PubMed | ("Heart Failure"[Mesh] OR "Heart Failure"[Title/Abstract]) AND ("Death, Sudden, Cardiac"[Mesh] OR "Sudden Cardiac Death"[Title/Abstract]) AND ("Risk Assessment"[Mesh] OR "Risk Prediction"[Title/Abstract] OR "Risk Model"[Title/Abstract] OR "Risk Score"[Title/Abstract]) | 669 |
| Web of Science | TS=("Heart Failure" ) AND TS=("Sudden Cardiac Death") AND TS=("Risk Assessment" OR "Risk Prediction" OR "Risk Model" OR "Risk Score") | 192 |
| Embase | #1 'heart failure':ti,ab  #2 'heart failure'/exp  #3 'sudden cardiac death':ti,ab  #4 'sudden cardiac death'/exp  #5 'risk model'/exp  #6 'risk prediction':ti,ab  #7 'risk model':ti,ab  #8 'risk score':ti,ab  #9 #1 OR #2  #10 #3 OR #4  #11 #5 OR #6 OR #7 OR #8  #12 #9 AND #10 AND #11 | 755 |
| The Cochrane Library | #1 "Heart Failure"  #2 MeSH descriptor:[ Heart Failure] this term only  #3 "Sudden Cardiac Death"  #4 MeSH descriptor:[ Death, Sudden, Cardiac] this term only  #5 "Risk Assessment" OR "Risk Prediction" OR "Risk Model" OR "Risk Score"  #6 MeSH descriptor:[ Risk Factors] this term only  #7 #1 OR #2  #8 #3 OR #4  #9 #5 OR #6  #10 #7 AND #8 AND #9 | 207 |
| Google Scholar | intitle:"Heart Failure" AND intitle:"Sudden Cardiac Death" AND (intitle:"Risk Assessment" OR intitle:"Risk Prediction" OR intitle:"Risk Model" OR intitle:"Risk Score") | 24 |
| Sinomed | "Heart Failure"[Common Fields: Intelligent] AND "Prediction"[Common Fields: Intelligent] AND "Cardiac Sudden Death"[Common Fields: Intelligent] | 41 |
| CNKI | "Heart Failure"[Abstract Fuzzy] AND "Prediction"[Abstract Fuzzy] AND "Cardiac Sudden Death"[Abstract Exact] | 80 |
| Wanfang | "Heart Failure"[Abstract Fuzzy] AND "Prediction"[Abstract Fuzzy] AND "Cardiac Sudden Death"[Abstract Fuzzy] | 70 |
