# Supplementary material for: Risk factors for sudden cardiac death in heart failure: A systematic review and meta-analysis of risk factors derived from prediction models
Source: Medicine (Baltimore). 2026 Aug 28;105(35):e50378. doi: 10.1097/MD.0000000000050378 (PMC13529164; doi:10.1097/MD.0000000000050378)
Supplement: Supplementary file 5 [file medi-105-e50378-s004.doc]

Supplemental Table 3. Meta-analysis for other risk factors of SCD.

| Risks of SCD | HR (95%CI) | I^2^(%) | Statistical method | P value |
| --- | --- | --- | --- | --- |
| age | 1.05 (1.03-1.06) | 0 | A | ＜0.0001 |
| creatinine | 1.97 (1.32-2.95) | 58 | B | 0.001 |
| diabetes mellitus | 1.61 (1.27-2.05) | 0 | A | ＜0.0001 |
| gender | 1.87 (1.49-2.35) | 0 | A | ＜0.0001 |
| heart rate | 1.02 (0.99-1.04) | 53 | B | 0.27 |
| ischemic aetiology | 1.57 (1.24-2.00) | 21 | A | 0.0002 |
| potassium | 1.53 (1.24-1.89) | 0 | A | ＜0.0001 |
| eGFR | 0.98 (0.96-1.00) | 73 | B | 0.07 |
| haemoglobin | 0.89 (0.83-0.96) | 19 | A | 0.0017 |
| sodium | 0.96 (0.93-0.99) | 0 | A | 0.0044 |

SCD: sudden cardiac death A: common model; B: random model; eGFR: estimated glomerular filtration rate
